# Frontier studies on fatigue, autonomic nerve dysfunction, and sleep-rhythm disorder

**DOI:** 10.1007/s12576-015-0399-y

**Published:** 2015-09-29

**Authors:** Masaaki Tanaka, Seiki Tajima, Kei Mizuno, Akira Ishii, Yukuo Konishi, Teruhisa Miike, Yasuyoshi Watanabe

**Affiliations:** Department of Physiology, Osaka City University Graduate School of Medicine, 1-4-3 Asahimachi, Abeno-ku, Osaka 545-8585 Japan; Hyogo Children’s Sleep and Development Medical Research Center, Hyogo Rehabilitation Centre, Central Hospital 1070 Akebono-cho, Nishi-ku, Kobe, Hyogo 651-2181 Japan; RIKEN Center for Life Science Technologies, 6-7-3 Minatojima-minamimachi, Chuo-ku, Kobe, Hyogo 650-0047 Japan

**Keywords:** Autonomic nervous system, Central nervous system, Circadian rhythm, Fatigue, Sleep

## Abstract

Fatigue is defined as a condition or phenomenon of decreased ability and efficiency of mental and/or physical activities, caused by excessive mental or physical activities, diseases, or syndromes. It is often accompanied by a peculiar sense of discomfort, a desire to rest, and reduced motivation, referred to as fatigue sensation. Acute fatigue is a normal condition or phenomenon that disappears after a period of rest; in contrast, chronic fatigue, lasting at least 6 months, does not disappear after ordinary rest. Chronic fatigue impairs activities and contributes to various medical conditions, such as cardiovascular disease, epileptic seizures, and death. In addition, many people complain of chronic fatigue. For example, in Japan, more than one third of the general adult population complains of chronic fatigue. It would thus be of great value to clarify the mechanisms underlying chronic fatigue and to develop efficient treatment methods to overcome it. Here, we review data primarily from behavioral, electrophysiological, and neuroimaging experiments related to neural dysfunction as well as autonomic nervous system, sleep, and circadian rhythm disorders in fatigue. These data provide new perspectives on the mechanisms underlying chronic fatigue and on overcoming it.

## Introduction

Fatigue is an indispensable bio-alarm, without which we might drop into an unrecoverable exhaustive state, and in the most severe case, even die, referred to in Japanese as “Karoshi”. It is likely that all people have experienced fatigue to some extent, and we know that this sensation decreases the efficiency of our daily tasks or studies. Thus, it is of great value to modern society for scientists to analyze the causes of fatigue and develop methods to quantify fatigue, with the goal of developing methods or therapies to afford better recovery from, and perhaps even avoidance of, severe chronic fatigue.

The following achievements had been made through previous projects: (1) elucidation of the brain regions and their neurotransmitter systems responsible for the fatigue sensation and chronic fatigue; (2) development of a variety of methods and scales to quantitatively evaluate the extent of fatigue; (3) development of animal models based on different causes of fatigue; (4) elucidation of molecular/neural mechanisms of fatigue in humans and animals; and (5) invention of various methods or therapies to treat chronic fatigue and chronic fatigue syndrome (CFS). Many researches are now promoting large-scale research projects on the molecular/neural mechanisms of fatigue and chronic fatigue and are also attempting to develop the therapeutics and remedies to improve the fatigued state. Such solutions are expected to provide a better quality of life for fatigued individuals. Here, we review data primarily from behavioral, electrophysiological, and neuroimaging experiments related to neural dysfunction as well as autonomic nervous system, sleep, and circadian rhythm disorders in fatigue. These data provide new perspectives on the mechanisms underlying chronic fatigue and on overcoming it.

## Fatigue and changes in autonomic function (Fig. 1)

### Autonomic function analysis

Frequency analyses for R–R interval variation of the electrocardiogram (ECG) or a–a interval variation of plethysmography (APG) are used with the maximum entropy method and fast Fourier transformation [1–3]. For frequency analyses, the low-frequency power component (LF) was calculated as the power within a frequency range of 0.04–0.15 Hz, and the high-frequency power component (HF) was calculated as that within a frequency range of 0.15–0.4 Hz. HF is vagally mediated [4–6], whereas LF originates from a variety of sympathetic and vagal mechanisms [4, 7]. The ratio of LF power component/HF power component (LF/HF ratio) is considered to represent sympathetic activity [8].

**Fig. 1:**
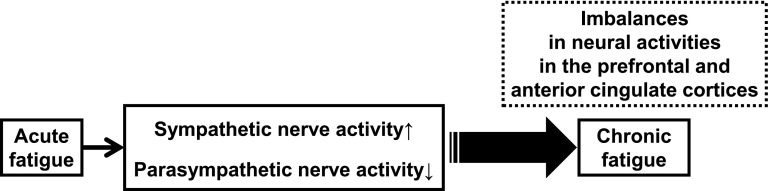
Autonomic function can be evaluated by electrocardiography or plethysmography. Frequency analysis of the data obtained from electrocardiography and accelerated plethysmography have revealed that enhanced sympathetic nerve activity based on a decrease in parasympathetic nerve activity is common in acute, sub-acute, and chronic fatigue. Alteration of autonomic function may result in changes and imbalances in neural activities in the central autonomic network of the brain, including the prefrontal and anterior cingulate cortices. Several interventions for the alleviation of fatigue are effective in decreasing sympathetic nerve activity. Evaluation of autonomic function contributes to investigating the effects of interventions for recovery from fatigue

### Acute fatigue and autonomic function

Acute fatigue in healthy individuals can be induced by loading lengthy mental tasks, such as the 2-back task (working memory task), advanced trail making test (switching attention), and the kana pick-out test (KPT; divided attention), for 30 min to 8 h [9–11]. As for autonomic function in the acute fatigue condition, decreased parasympathetic activity (low value of HF) and increased sympathetic activity (high value of LF/HF ratio) have been observed in healthy volunteers following a 30-min series of fatigue-inducing mental tasks [9, 10]. After a prolonged cognitive load for 8 h, corresponding to a normal workday, we found that sympathetic hyperactivity (high value of LF/HF ratio) based on decreased parasympathetic activity (low value of HF) was positively correlated with subjective fatigue level as evaluated by the visual analogue scale [11]. These findings indicate that acute mental fatigue is characterized by an increase in sympathetic nerve activity and a decrease in parasympathetic nerve activity.

### Sub-acute fatigue and autonomic function

Sub-acute levels of fatigue can be evaluated using Chalder’s fatigue scale, a paper-and-pencil questionnaire [12]. A Japanese version has also been developed, and the reliability and validity of the Japanese version to evaluate the severity of daily fatigue have been confirmed previously [13, 14]. Levels of sympathetic nerve activity (LF and LF/HF ratio) and parasympathetic nerve activity (HF) of healthy adults have been positively and negatively associated, respectively, with scores on Chalder’s fatigue scale [10]. These findings indicate that enhanced sympathetic nerve activity based on decreases in parasympathetic nerve activity is common in the acute and sub-acute fatigue conditions.

### Chronic fatigue and autonomic function

CFS is a disease characterized by chronic, profound, disabling, and unexplained fatigue [15]. Fatigue-related alterations of autonomic nervous system activities have been reported in adults with CFS [16–18]. Decreased parasympathetic nerve activity and increased sympathetic activity have also been observed in patients with CFS. Yamaguti et al. [18] reported that the sympathetic hyperactivity level of patients with CFS was dependent on the severity of symptoms as evaluated by a performance status test. Not only adults with CFS but also children and adolescents with CFS have been observed to have sympathetic hyperactivity based on decreased parasympathetic nerve activity [19]. These findings indicate that sympathetic hyperactivity based on low parasympathetic nerve activity is common in acute, sub-acute, and chronic fatigue.

### Autonomic alterations as mechanisms of fatigue

In terms of autonomic nerve function, the central autonomic network that controls sympathetico-vagal balance consists of the orbitofrontal cortex (OFC), medial prefrontal cortex (PFC), anterior cingulate cortex (ACC), insular cortex (IC), amygdala, bed nucleus of the stria terminalis, hypothalamus, periaqueductal gray matter, pons, and medulla oblongata [20, 21]. The ACC plays a particularly crucial role in the central control of sympathetico-vagal balance [22]. There are anatomical and functional connections between the dorsolateral PFC (DLPFC) and medial PFC, including the ACC and the OFC [23–26]. Sympathoexcitatory subcortical threat circuits are normally under the inhibitory control of the medial PFC [27–29].

In an experimental setting, acute mental fatigue of healthy volunteers has been produced by a prolonged load of executive function tasks such as working memory and attention control tasks [9–11]. Several studies using functional magnetic resonance imaging (fMRI) have reported that during prolonged mental tasks lasting 1–2 h, activation of the brain regions related to mental task processing was gradually reduced [30, 31]. During fatigue-inducing mental tasks, the PFC, including the ACC, which is associated with the processing of executive functions such as verbal working memory, visuospatial working memory, and divided attention in the 2-back test, advanced trail making test, and KPT, respectively, may be continuously activated [32]. However, these prefrontal activities may be gradually reduced with loading time. As with the medial OFC, this region is associated with fatigue sensation [33]. The medial OFC was shown to exhibit a positive correlation in activity with the subjective sensation of fatigue, measured immediately after each positron emission tomography (PET) scan with H_2_^15^O [a probe for regional cerebral blood flow (rCBF)] for 1.5 h. [33]. These results suggest that acute mental fatigue induces changes and imbalances in neural activities of the regions involved in the central autonomic network; thus, it is difficult to control the inhibitory capacity of the sympathoexcitatory response. Evidence such as lowered cerebral activity in the PFC during fatigue-inducing tasks [34] and a bilateral reduction of gray-matter volume in the prefrontal cortices of patients with CFS [35] suggests that individuals with CFS may exhibit anatomical and functional alterations in the PFC. Because the role of the PFC is essential in active tonic inhibition of sympathoexcitatory threat circuits, such alterations in the PFC seen in patients with CFS could be expected to lead to a decrease in parasympathetic drive, defaulting to a sympathetically driven system. Therefore, it is possible that an accumulation of mental fatigue (sub-acute mental fatigue and chronic fatigue) in healthy people induces a prolonged deterioration of autonomic activity through anatomical and functional alterations of the PFC.

### Application of autonomic function in clinical and industrial settings

To measure autonomic nerve function and fatigue levels, both in clinical and industrial settings, better experimental designs, including briefer mental tasks and more sensitive measurement methods for detecting changes in sympathico-vagal balance, are needed. We designed a 16-min period of ECG measurement during a resting state before the KPT, with participants’ eyes open for 3 min and closed for 3 min, during the KPT for 4 min, and during a resting state after the KPT with their eyes open for 3 min and closed for 3 min. We investigated the correlation between fatigue sensation and alterations of autonomic nerve activity using this protocol to perform the KPT in healthy volunteers [36]. The KPT is frequently used as a fatigue-evaluation test in both children and adults [19, 36, 37]. A baseline fatigue sensation, measured by a visual analogue scale before the experiment, was associated with an increase in sympathetic nerve activity (LF/HF ratio) during the KPT. The LF/HF ratio during the post-KPT resting state with eyes open tended to be greater than the ratio during the KPT and was correlated with fatigue sensation. Fatigue sensation was correlated negatively with parasympathetic nerve activity (HF) during the post-KPT rest period with eyes open. These results suggest that a brief mental task can be used to evaluate changes in autonomic nerve activity with fatigue if the eyes-open condition is used. Therefore, the methods described here are useful for assessing the association between fatigue sensation and autonomic nerve activity involved in fatigability using a brief cognitive test.

### Anti-fatigue and autonomic function

To prevent healthy individuals from suffering from chronic fatigue, early intervention and evaluation of the effects of intervention are important. For this purpose, autonomic functions may be useful as objective physiological markers for acute and chronic fatigue. We previously studied anti-fatigue effects on acute mental fatigue of healthy adults during bathing normally and during bathing with water containing micro-bubbles. A sensation of reduced fatigue, as detected by the visual analogue scale after the fatigue-inducing mental task for 4 h, was negatively associated with sympathetic nerve activity (LF/HF ratio) only in the micro-bubble bathing condition. These findings suggest that micro-bubble bathing is effective in preventing an increase in acute mental fatigue [38]. An equivalent mixture of 0.03 % *cis*-3-hexenol and 0.03 % *trans*-2-hexenal diluted with triethyl citrate is known to have a healing effect on psychological damage caused by stress. The behavioral studies described above in humans and monkeys revealed that hexenol/hexenal prevented the prolongation of reaction time caused by fatigue [39]; the ACC was activated by the fragrance of hexenol/hexenal. This finding suggests that an increase in rCBF using H_2_^15^O as detected by PET in the ACC caused by the fragrance of hexenol/hexenal may contribute to its healing effects observed in monkeys [40]. In a human study, the task performance of healthy volunteers did not decrease and sympathetic nerve activity did not increase by sniffing the aroma of hexenol/hexenal during prolonged performance of the advanced trail making test, indicating that this fatigue-alleviation effect might occur via an autonomic function, such as a healing effect on the sympathetic nervous system caused by stimulating the activity of the central autonomic network, especially the cingulate cortex [39].

Sympathetic hyperactivity based on a decrease in parasympathetic nerve activity is common in acute, sub-acute, and chronic fatigue. This autonomic function alteration is related to a decrease in the brain function of the central autonomic network. To prevent the accumulation of fatigue, interventions on recovery from fatigue via normalization of sympathetic hyperactivity are important. Findings from studies done by a collaboration of industry, academia, and government may lead to the development of an anti-fatigue solution.

## Role of sleep and circadian rhythm in fatigue recovery (Fig. 2)

### Sleep features in individuals with CFS or chronic fatigue state

CFS is a medically unexplained disabling illness characterized by persistent relapsing fatigue of at least 6 months’ duration with low activity levels. Post-exertion malaise, neurocognitive dysfunction, infection-related symptoms, autonomic dysfunctions, and sleep disturbances are also major features of CFS. Six studies reported no CFS specific findings in polysomnography (PSG) or actigraphy in contrast with CFS patients’ significant sleep complaints [41–46]. Fatigue or pain was well correlated with sleep disturbances and daily activity in patients with CFS or a CFS-related disorder, such as fibromyalgia [43, 47–49]. Interestingly, there was a weak correlation between fatigue score and sleepiness score in individuals without fatigue-related disorders [50]. Under ambulatory monitoring conditions using home-based PSG or actigraphy, patients with CFS showed significant longer bedtime sleep, longer awake time after sleep onset, and less efficient sleep [51–55]. Individuals with CFS had a high incidence of undiagnosed primary sleep disorders [56, 57]. This result strikes a note of warning for physicians. Eight studies reported objective abnormalities in patients with CFS in terms of respiratory index, electroencephalographic power spectrum, and sleep-awake switching dynamics [58–65]. Gotts et al. showed that patients with CFS were divided into four clusters by sleep features based on hierarchical cluster analysis [66]. Those dynamics-based and cluster analysis results may reveal the heterogeneity of CFS pathophysiology and a new viewpoint regarding an analytical approach. It was also noted that total Pittsburgh sleep quality index score might not be suitable for patients with CFS [67, 68]. There were no significant actigraphic or polysomnographic differences between patients with CFS and a control group [49, 65, 68].

**Fig. 2:**
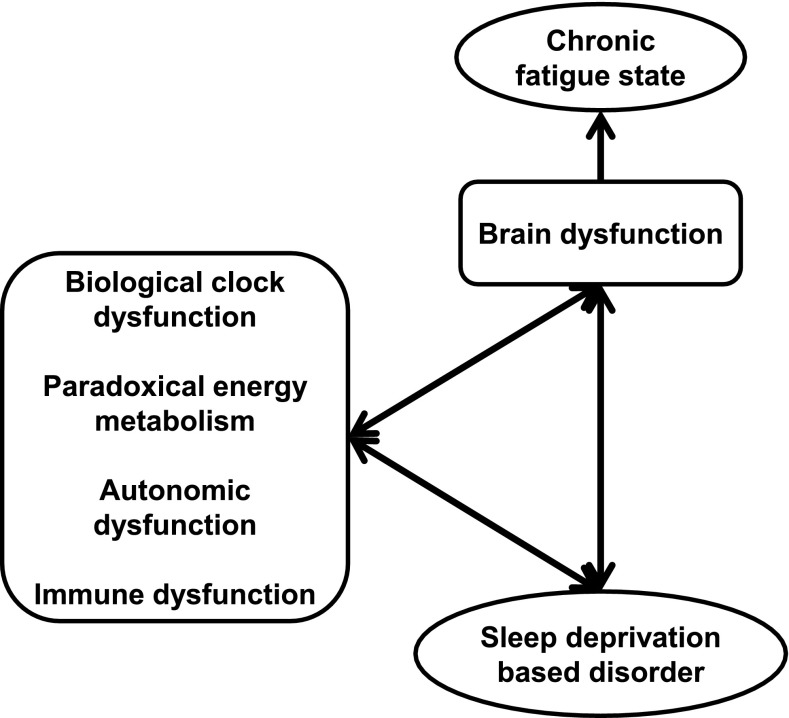
Chronic fatigue state induced by sleep deprivation. Accumulation of sleep deprivation caused brain dysfunction with/without biological dysfunctions. Fundamental biofunctions (biological clock, energy metabolism, autonomic activity, and immune system), sleep disorders, and brain functions interacted with each other and created negative chains. Chronic fatigue state was observed as the output of brain dysfunctions

### Physical exercise effects on CFS

To examine the effect of exercise on post-exertional exacerbations, Ohashi et al. performed actigraphic circadian analysis for approximately 1 week before and after performing a maximal treadmill test in the patients with CFS and healthy controls. In that study, lengthening the mean circadian period induced by one period of exercise remained several days in patients with CFS [69]. No significant PSG index change was observed after exercise load [70]. However, in terms of sleep stage transition dynamics, significant decreases in sleep stage continuity caused by exercise were revealed [71].

### Circadian aspect of sleep and fractal features of diurnal and nocturnal activity in CFS

Dim-light, melatonin-onset delay was observed repeatedly in patients with CFS [72, 73, 75]. Dissociation of body-temperature and melatonin secretion circadian rhythms were also found in patients with CFS [74]. Fluctuations of circadian melatonin secretion and core body temperature were reported in patients with CFS [72, 75, 76]. From a therapeutic point of view, abnormal circadian oscillation of core body temperature, plasma melatonin, cortisol, and β-endorphin levels were improved by treatment with methylcobalamin and melatonin in a patient with pediatric CFS [72].

Ohashi et al. found a significant diurnal rest-action structure change in patients with CFS [77]. The fractal feature change of physical activity was confirmed in Japan [78], and also in patients with pediatric CFS [79].

### Clock gene expression in pediatric CFS

We reported that accumulation of sleep deprivation altered biological rhythm can lead to pediatric CFS [72, 76, 79]. Based on these findings, we hypothesized that biological clock modulation caused pediatric chronic fatigue pathophysiology. To address this hypothesis, Takimoto et al. performed clock gene expression analysis in patients with pediatric CFS [80]. That study suggested that the monitoring of human clock genes in whole blood cells, which may be functionally important for the molecular control of the circadian pacemaker as well as in the suprachiasmatic nucleus, might be useful for the evaluation of internal synchronization.

### Nocturnal autonomic nerve activity in CFS

Less nocturnal vagal tone in patients with CFS has been reported frequently [13, 17, 81, 87]. Correlation between fatigue severity and a diurnal sympathetic activity was also found [82]. Diurnal sympathetic hyperactivity may cause an accumulation of the fatigue state and decreased vagal tone during sleep may cause unrefreshing sleep.

### Brain imaging and cognitive impairment in CFS

A significant decrease of blood flow in the frontal, temporal, and occipital lobes, and a decrease of metabolic levels in the frontal lobe were observed in patients with pediatric CFS [83, 84]. This suppressed cerebral blood flow and energy metabolism may be relevant to cognitive dysfunction. Recently, significant progress has been made in the field of brain functional imaging. Ideas for connecting and using these findings are expected in the near future.

In patients with pediatric CFS, abnormally prolonged P300 latency to target stimuli might be associated with learning disability, and abnormally exaggerated P300 amplitude to non-target stimuli might be associated with hypersensitivity [19]. Those subtypes, divided by cognitive function, were correlated with fatigue state severity. In contrast to pediatric findings, napping, particularly in the afternoon, was associated with poorer cognitive functioning and more daytime sleepiness in adult patients with CFS [85]. These findings have clinical implications for symptom management strategies.

### Oxidative stress and cytokines in CFS

Methemoglobin was found to be the major component correlated with variation in symptom expression in patients with CFS; symptoms included fatigue, musculoskeletal symptoms, pain, and sleep disturbances. Variations in levels of malondialdehyde and 2,3-diphosphoglycerate were also correlated with variations in cognitive symptoms and sleep disturbances [86]. These results suggest that oxidative stress due to excess free radical formation is a contributor to the pathology of CFS and is associated with symptom presentation.

mRNA expression of cytokines was suppressed under the condition of sleep disturbance in patients with pediatric CFS [87]. A major hypothesis regarding the cause of CFS is immune dysregulation such as upregulation of proinflammatory cytokines. There are discrepancies between results of immunological studies in patients with pediatric CFS and a major hypothesis in adult CFS pathogenesis. Nakamura et al. reported that physical exertion or sleep deprivation did not produce clinically significant upregulation of proinflammatory cytokines [88]. This latter result complicates the discrepancies found. Further studies will be needed.

### Peripheral energy metabolism in pediatric CFS

Fatigability in patients with CFS seems to be based on down-regulation of energy metabolism clinically. To address this clinical question, Iwatani et al. performed the oral glucose tolerance test to measure glucose metabolism in patients with pediatric CFS [89]. Results indicated that glucose dysregulation in patients with pediatric CFS appeared to be caused by emotional distress. Multiple factors, including autonomic nervous system disorders, derangement of neuropeptides in the hypothalamus, and hormonal imbalances, may also affect glucoregulatory metabolism, predisposing these patients to hyperglycemia. They concluded that the glucoregulatory system compensates for decreased blood flow to the brain by increasing blood glucose concentrations, thereby providing sufficient glucose as the primary energy source used during normal brain metabolism.

### Role of sleep and circadian rhythm in fatigue recovery

PSG assessment based on conventional approaches show no useful information to assess the pathophysiology of CFS. Physiological data, for example, electroencephalograms (EEGs), electrocardiograms, physical activity, and core body temperature, should be assessed by new approaches such as dynamic system analysis.

Sleep deprivation has a high impact on chronic fatigue pathophysiology, especially in childhood. Accumulation of sleep deprivation alters the biological rhythm. We have reported that alterations in biological rhythm increase circadian biological dysfunctions, for example, endocrine, energy metabolism, autonomic activity, and neurocognitive dysfunctions.

It is still a challenge to recover from CFS today. Prevention of CFS is the only way to minimize the social impact of this disorder. The relationship between chronic fatigue pathogenesis and sleep deprivation is apparent. Therefore, Miike, the director of Hyogo Children’s Sleep and Development Medical Research Center (HCSDMRC), and his colleagues keep challenging healthcare providers to prevent children’s sleep deprivation via sleep education not only in schools but also by region. Five years of intervention has been shown to prevent school non-attendance completely in some areas. However, prevention of school non-attendance is not equal to prevention of pediatric CFS. Miike proposes to extend this ‘sleep well action’ to infants and their families. The issue then becomes identifying the risk factors of future sleep disturbances for infants. The key was in the HCSDMRC clinical experiences. All infants who visited the HCSDMRC clinic due to sleep disturbances were irritable (78.6 %) or sluggish (21.4 %) in the neonatal period. Interestingly, 66 % of those who were sluggish changed to the irritable state in the first year of life. As the result of our ongoing clinical research, we hypothesize that those unusual states in the neonatal period are early predictors of autism spectrum disorder. Based on clinical evidence, we propose that irritable or sluggish newborns should be followed by medical specialists of pediatric sleep medicine.

## Neural mechanisms of fatigue

### Acute physical fatigue and the central nervous system

When subjects are physically fatigued, they increase voluntary efforts to increase the motor output from the primary motor cortex (M1) to compensate until the task requires a maximal effort [90]. Using an electrophysiological technique, a facilitation system to increase motor output from the M1 against physical fatigue was suggested [91]. While participants were performing a maximum voluntary contraction (MVC), a short-latency excitatory electromyography (EMG) response in the M1 increased, indicating increased excitation. Because this change was unaffected by the manipulation of afferent inputs, this was considered to be the result of an enhanced voluntary drive to the M1 via activation of the facilitation system [91].

This facilitation system has also been described in neurophysiological and neuroimaging studies, during fatigue-inducing submaximal physical task trials. Increased activations (area and intensity) in the contralateral M1 have been observed in EEG [92, 93], PET [94, 95], and fMRI [96–99] studies, which matched the EMG signals [96] until the activations reached the maximum level. The increase in the contralateral M1 suggests recruitment of inactive neurons, and this recruitment may result in an increase in the drive to the target muscles but can also activate other non-target muscles. Because increases in intensity across EEG, PET, and fMRI studies correlate with excitatory changes in the local field potentials [100, 101], the increase in intensity suggests increased excitatory input and sensory processing, and subsequent enhanced corticomotor drive to the target muscles by the contralateral M1.

The facilitation system may include the ipsilateral M1. While participants performed submaximal voluntary contractions, the fMRI activation volume in the ipsilateral M1 exhibited a steady increase [96]. Because 7–8 % of M1 neurons are associated with ipsilateral movements [102], additional recruitment of the cortical motoneurons from the ipsilateral side may work to compensate for central nervous system fatigue.

In addition to the contralateral and ipsilateral M1, activation of other brain regions has been shown during submaximal physical tasks, and these areas may be involved in the facilitation system. EEG signals showed location shifts toward the ipsilateral, anterior, and inferior sides, indicating augmentation in the ipsilateral sensorimotor, prefrontal, orbitofrontal, and anterior cingulate areas [93], and increased EEG responses in the bilateral supplementary motor area (SMA) have been observed [92]. Similar activation patterns have been shown in rCBF responses assessed using PET, magnetic responses using magnetoencephalography (MEG), and blood oxygen level-dependent (BOLD) responses using fMRI. Increases in the rCBF response in the bilateral SMA, ACC, PFC, basal ganglia (BG), and thalamus (TH) [94, 95], in the magnetic responses in the ipsilateral sensorimotor area and PFC [103–105], and in the BOLD responses (area and intensity) in the bilateral SMA, ACC, and PFC [96, 99] were shown during the time course of submaximal motor tasks.

Based on the results of these electrophysiological and neuroimaging studies and the knowledge of neural interconnections [106], we proposed a neural pathway or circuit that constitutes the facilitation system to increase the motor output from the M1 to compensate for the effects of physical fatigue: the neural circuit or re-entrant loop that interconnects the limbic system, BG, TH, OFC, PFC, ACC, SMA, and M1, constitutes the facilitation system and an increase in the motivational input to this facilitation system enhances the SMA and then M1 to increase the motor output to muscles via the spinal cord [107].

### Acute mental fatigue and the central nervous system

The facilitation system of mental fatigue has also been described in behavioral, electrophysiological, and neuroimaging studies of fatigue-inducing submaximal mental tasks [9, 108–118]. Some reports suggest that cognitive task performance is maintained during the mental task trials, even when participants are fatigued [9, 108–110, 113, 115]. For example, cognitive task performance assessed by reaction time and accuracy did not change over time in a fatigue-inducing mental task session [9, 110, 113, 115]. The advanced trail making test (ATMT) [119] was used to determine whether participants were mentally fatigued. In the ATMT, circles numbered from 1 to 25 are randomly located on the display of a personal computer, and participants are required to use a computer mouse to click the center of the circles in sequence, starting with circle number 1. The number of errors on the ATMT and the subjective level of mental fatigue was increased after the fatigue-inducing mental task trials. These findings demonstrate that participants were mentally fatigued after the mental task, and suggest that cognitive performance was not altered by a compensatory mechanism, that is, the mental facilitation system [113].

As described previously, frequency domain analyses of electrocardiography R–R wave intervals can be used to evaluate activity of the sympathetic nervous system [8], and findings indicate that the sympathetic nervous system is active during a fatigue-inducing mental task session [9, 11]. Increased motivation or mental effort has been associated with increased sympathetic nervous system activation [120, 121]; therefore, the increase in sympathetic nervous system activity during the mental task session may reflect an increase in the contribution of motivation or mental effort to the maintenance of cognitive task performance in the presence of mental fatigue. Considerable evidence supports this assumption; for example, an increased level of motivation resulted in an improvement of cognitive task performance during a fatigue-inducing mental task [30].

Oscillatory brain rhythms are considered to originate from synchronous synaptic activities of a large number of neurons [122]. Synchronization of neural networks may reflect integration of information processing, and continuously interacting dynamic neural networks are assessed through the synchronization of oscillations at particular time–frequency bands [123]. Such synchronization processes can be evaluated using MEG time–frequency analyses. Alpha-band (8–13 Hz) power in the frontal cortex was lower after the fatigue-inducing mental task session than before [113]. Large-scale, rhythmic oscillations in brain activity at alpha-band frequencies are generated by interactions between thalamo-cortical neurons and GABAergic (*γ*-aminobutyric acid) cells in the thalamic reticular nucleus [124, 125]. Therefore, suppressed spontaneous alpha-band power, that is, desynchronization due to intrinsic events in the frontal cortex caused by mental fatigue suggests an overactivation of the thalamic-frontal circuit. Considering that task performance was maintained during the fatigue-inducing mental task trials, this thalamic-frontal circuit is a candidate neural substrate for the mental facilitation system related to motivation or mental effort [113].

A physical task with moderate intensity improved cognitive function and enhanced functional near-infrared spectroscopy response in the frontal area [126]. This finding implies that the physical facilitation system shares common neural substrates with the mental facilitation system; that is, activation of the physical facilitation system may enhance the mental facilitation system through activation of common neural networks, including the frontal area [127]. The physical facilitation system is a re-entrant neural circuit that interconnects the limbic system, BG, TH, OFC, DLPFC, ACC, SMA, and M1 [107]. The results of studies using event-related potentials indicate that evaluation of predicted rewards and potential risks of actions are central to mental fatigue, and the evaluation system was considered to consist of a neural circuit that interconnects the limbic system, BG, TH, OFC, DLPFC, and ACC [128]. In addition, resting rCBF assessed using fMRI in the TH and frontal cortex was positively associated with cognitive task performance during mental fatigue [129]. Hence, the mental facilitation system may be a neural circuit that interconnects the limbic system, BG, TH, and frontal cortex, and an increase in motivational input to this facilitation system may activate the system and compensate for the effects of mental fatigue [130].

### Chronic fatigue and the central nervous system

Dysfunction of the facilitation system in subjects with chronic fatigue has been indicated through alterations of cognitive function. During Stroop trials, color and word dimensions activate the associated responses, resulting in a conflict between the activated responses and an increased likelihood of error [131]. This conflict is proposed to activate a conflict monitor in the ACC, which in turn engages the control function in the DLPFC. The engagement of the DLPFC increases attention on subsequent trials, resulting in improved performance [132]. Because the level of chronic fatigue was positively associated with the error rate of the Stroop trials [115], chronic fatigue might cause deterioration in response inhibition through impaired functions in the ACC and/or DLPFC.

It was proposed that fatigue associated with neurological disorders occurs due to a failure in the facilitation system [133, 134]. In a 2-[^18^F]fluoro-2-deoxy-d-glucose (FDG) PET study, subjects with fatigue from multiple sclerosis (MS) showed reduced FDG uptake in the brain regions involved in the striatal-thalamic-frontal circuit [135]. In MS patients without impaired hand function, greater fMRI responses relative to healthy participants have been reported during simple hand movements in the contralateral M1 [136, 137], while after fatigue-inducing handgrip trials, these MS patients did not show greater activation in this brain region although healthy participants showed greater activation [137]. These results can be interpreted as follows: dysfunction of the facilitation system contributes to chronic fatigue in these patients. As for CFS, even a minor activity leads to significant worsening of fatigue [15], although these patients have normal or near-normal aerobic capacity [138] and muscle function [139]. Patients with CFS showed anatomic and/or metabolic impairments in the brain regions involved in the facilitation system, that is, the BG [140], OFC [35, 141–143], PFC [141, 142, 144], and ACC [141, 142]. Thus, dysfunction of the facilitation system seems to contribute to the pathophysiology of chronic fatigue-related diseases or syndromes.

Enhancement of the facilitation system can cause neural activation (higher level of and wider area of neural activation) [23, 29, 145–148] and induce the release of large quantities of excitatory amino acids, such as glutamate and aspartate. Released glutamate binds to different receptors; the main one being the *N*-methyl-d-aspartate subtype, whose activation causes mobilization of free cytosolic calcium. Excessive intracellular calcium concentrations lead to over-activation of certain calcium-dependent enzymes, resulting in the generation of proinflammatory cytokines, chemokines, inflammatory mediators, and reactive oxygen and nitrogen species to cause oxidative stress, inflammation, and energy deficiency [149–158]. Hence, repetitive and prolonged overwork and/or stress without sufficient recovery to enhance the facilitation system seem to induce oxidative stress, inflammation, and energy deficiency in the central nervous system and cause neural damage followed by dysfunction of the facilitation system. Chronic activation of sustained oxidative stress [69], inflammation [159], and secondary mitochondrial dysfunction and impaired energy metabolism [160] in the central nervous system are also considered to be involved in the pathophysiology of CFS.

**Fig. 3:**
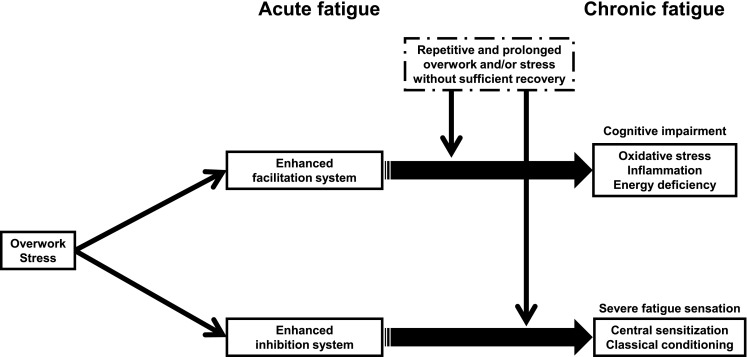
Hypothetical model of the development of chronic fatigue. When subjects are acutely fatigued through overwork and/or stress, they progressively increase their voluntary effort to maintain their performance to compensate for acute fatigue until the work requires a maximal effort. At that point, the facilitation system in the central nervous system is activated to overcome acute fatigue. The facilitation system consists of a re-entrant neural circuit that interconnects the limbic system, basal ganglia, thalamus, orbitofrontal cortex, prefrontal cortex, and anterior cingulate cortex, and a motivational input activates this system. In addition, as subjects become acutely fatigued, an alarm signal to take a rest (inhibitory system) is activated to avoid further fatigue. The inhibition system consists of a neural pathway that involves the insular and posterior cingulate cortices. After repetitive and prolonged overwork and/or stress that activates the facilitation system without sufficient recovery, the facilitation system dysfunctions, through neural damage to it caused by oxidative stress, inflammation, and energy deficiency. Subjects express impaired information processing in the central nervous system. In addition, repetitive and prolonged overwork and/or stress cause central sensitization and classical conditioning of the inhibition system. This conditioned inhibition system occurs in subjects with chronic fatigue, resulting in a long-lasting alarm signal to take a rest and a severe sustained fatigue sensation and functional disabilities

Based on these findings, we present here a hypothetical model of developing chronic fatigue (Fig. 3) [16, 161–165]. When subjects are acutely fatigued through overwork and/or stress, they progressively increase their voluntary effort to maintain their performance to compensate for acute fatigue until the work requires a maximal effort; at that point, the facilitation system in the central nervous system is activated (intensity and area) to overcome acute fatigue. The facilitation system consists of a re-entrant neural circuit that interconnects the limbic system, BG, TH, OFC, PFC, and ACC, and a motivational input activates this system. In addition, as subjects become acutely fatigued, an alarm signal to take a rest (inhibitory system) is activated to avoid further fatigue. The inhibition system consists of a neural pathway that involves the IC and PCC. We propose that after repetitive and prolonged overwork and/or stress that activates the facilitation system without sufficient recovery, the facilitation system dysfunctions, through neural damage to it caused by oxidative stress, inflammation, and energy deficiency. Subjects express impaired information processing in the central nervous system. In addition, we propose that repetitive and prolonged overwork and/or stress cause central sensitization and classical conditioning of the inhibition system. This conditioned inhibition system occurs in subjects with chronic fatigue, resulting in a long-lasting alarm signal to take a rest and a severe sustained fatigue sensation and functional disabilities.

## Neural mechanisms of fatigue sensation

### Fatigue sensation

Fatigue sensation acts as a biological alarm to rest to maintain homeostasis and constitutes the inhibition system of fatigue. However, if the fatigue sensation is over-activated, such as occurs through classical conditioning and/or central sensitization, a decline in the performance of mental and physical activities may occur [165]. Therefore, it is important to understand the neural mechanisms of fatigue sensation and test whether fatigue sensation can be caused through classical conditioning and/or central sensitization.

An MEG study related to the mirror system of fatigue sensation has been reported [166]. Twelve healthy male volunteers participated in this study and viewed 80 pictures with fatigued facial expressions and those with neutral facial expressions in a randomized order. Because there have been several reports showing that seeing emotional changes in others activates the brain regions involved in experiencing similar emotions [167–173], it is hypothesized that the brain regions activated when they viewed the fatigued facial expressions may be candidate brain regions related to the neural mechanisms of fatigue sensation. In fact, the equivalent current dipole (ECD) in the PCC was observed in 9 of 12 participants, and the ECD in the IC was observed in 3 of 12 participants only when they viewed the fatigued facial expressions, suggesting that the PCC and the IC are the candidate brain regions related to the neural mechanisms of fatigue sensation [166]. Because it has been reported that the PCC is involved in self-reflection or self-monitoring [174–176], the neural substrates related to self-evaluation of the level of physical and mental fatigue were examined using MEG. Ten healthy male volunteers participated in a study that examined the neural substrates related to self-evaluation of the level of physical fatigue. When they self-evaluated the level of fatigue of their right hand, the ECD in the PCC was observed in 9 of 10 participants. On the other hand, when they directed their attention to their right hand as a control condition, the ECD in the PCC was observed in 2 of 10 participants. In addition, the intensity of the ECD in the PCC observed in relation to the self-evaluation of the level of physical fatigue was positively associated with the extent to which fatigue of the right hand was successfully evaluated. These results suggest that the activation in the PCC was related to the self-evaluation of the level of physical fatigue [177]. In the next study, the neural substrates related to self-evaluation of the level of mental fatigue were examined. Fourteen healthy male volunteers participated in this study. They performed 90 evaluation trials and 90 control trials in a randomized order. In the evaluation trials, they were asked to self-evaluate the level of their mental fatigue. The control trials were resting trials in which they were asked to do nothing. The ECD in the PCC was observed in 7 of 14 participants when they self-evaluated the level of their mental fatigue, although it was observed in only 1 of 14 participants when they performed the control trials, suggesting that the activation in the PCC was also related to self-evaluation of the level of mental fatigue [178]. It has been shown that the PCC is not only involved in the neural mechanisms of fatigue sensation but is also involved in the neural mechanisms of making decisions in the presence of fatigue [179]. If individuals do not rest, despite the signs of fatigue, they may experience overwork, which may be a starting point of chronic fatigue as discussed later. Therefore, the decision of whether or not to rest based on the level of fatigue is important. Fifteen healthy male volunteers participated in this study. They performed 1200 reverse Stroop test trials and were intermittently asked whether to take a rest or not to maintain task performance; neural activities related to making decisions to rest were assessed. When they made decisions to rest, a decreased 4–8 Hz band power was observed in the PCC, and this decreased 4–8 Hz band power in the PCC was positively associated with the subjective level of fatigue caused by performing the experiment. As for the IC, it has been reported that the IC is involved in mental effort evaluation in an fMRI study in which the participants rated their mental effort investment required for performing 1-, 2-, and 3-back tests [180]. These findings suggest that the PCC and IC are involved in the neural mechanisms of fatigue sensation to self-evaluate the level of fatigue, and that the PCC plays an important role in making decisions to take a rest in the presence of fatigue.

### Classical conditioning of fatigue sensation

It has been reported that classical conditioning related to fatigue took place in rats [181]. In this study, rats received paired conditioned and unconditioned stimuli. Feeding of a sucrose solution was used as a conditioned stimulus, and intraperitoneal injection of a synthetic double-stranded RNA, polyriboinosinic:polyribocytidylic acid (poly I:C), was used as a unconditioned stimulus. Because the poly I:C injection has been shown to be related to decreased spontaneous activity on the running wheel, injection of the poly I:C was used to make the rats fatigued [182–184]. After 4 days of this conditioning, the rats showed decreased spontaneous activity only when given the sucrose solution.

**Fig. 4:**
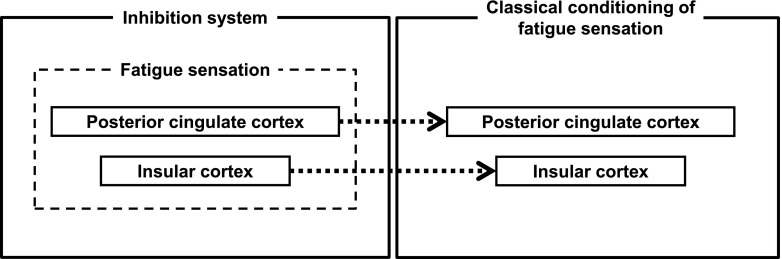
The brain regions involved in the neural mechanisms of fatigue sensation and those of classical conditioning of fatigue sensation. The neural mechanisms of fatigue sensation, which constitute the inhibition system of fatigue, include the posterior cingulate and insular cortices. The posterior cingulate and insular cortices are also involved in the neural mechanisms of the classical conditioning of fatigue sensation

As it is hypothesized that the fatigue sensation induced by classical conditioning can be a cause of chronic fatigue [165], it is important to examine whether the fatigue sensation can be classically conditioned in humans and to clarify the neural mechanisms related to the classical conditioning of fatigue sensation in case it occurs. In fact, it has been shown that mental and physical fatigue sensation can be classically conditioned in humans in experimental settings. Ten healthy male volunteers participated in a study that examined whether mental fatigue sensation can be classically conditioned [185]. On the first day, MEG was recorded for 6 min while listening to metronome sounds (first MEG session), and then participants performed the 2-back test for 60 min as a conditioning session. Because it has been reported that performing the 2-back test for 30 min induced significant levels of the fatigue sensation [9, 10], we started the metronome sounds 30 min after the 2-back test started. On the second day, MEG was recorded again while listening to the metronome sounds (second MEG session). The level of the fatigue sensation caused by the metronome sounds in the second MEG session was significantly higher than that in the first MEG session, suggesting that the classical conditioning of mental fatigue sensation took place. An ECD analysis was performed for the MEG data from eight participants because the MEG data from two participants were contaminated with magnetic noise and excluded from the analysis. The ECD in the IC was observed in 6 of 8 participants and that in the PCC was observed in 4 of 8 participants only in the second MEG session. Because these magnetic responses were observed only in the second MEG session, these magnetic responses were thought to be involved in the neural mechanisms of fatigue sensation related to the classical conditioning. The classical conditioning of physical fatigue sensation in humans has also been reported [186]. Eight healthy male volunteers participated in this study. The experimental design of this study was the same as the previous one except for the conditioning session: they performed a hand grip task for 10 min, and the metronome sounds were started 5 min after the task started. Similar to the previous study, fatigue sensation was caused by listening to the metronome sounds after the conditioning session and the ECD in the PCC was observed only in the second MEG session in all participants. These findings suggest that mental and physical fatigue sensation can be classically conditioned in humans in experimental settings, and that the PCC and IC seem to be involved in the neural mechanisms of classical conditioning related to mental and physical fatigue sensation. Taking these findings into consideration, the PCC and IC are involved in the neural mechanisms of inhibition systems and seem to play important roles in the pathophysiology of chronic fatigue (Fig. 4).

## Conclusion

Information related to the mechanisms underlying fatigue is still not complete. A major obstacle to this understanding involves limitations in our evaluation methods to understand the complex, dynamic, and interactive nature of fatigue. Several challenges, in particular advanced human behavioral, physiological, and neuroimaging studies, need to be met to obtain sufficient information to understand the mechanisms underlying fatigue.

The best treatment for any disease is to prevent the disease before it occurs. In this sense, based on the risk or predictive factors for a disease, early selection of a high-risk group and intensive preventive interventions for this group would be an efficient preventive strategy for the disease. In particular, the importance of developing individualized preventive strategies should be emphasized. If sufficient intervention is not performed, the disease will likely develop. However, it is difficult to select the best preventive method for chronic fatigue, as few data are available to predict future disease. It would thus be beneficial to differentially diagnose future disease for each subject based on the subject’s individual information, including symptomatic, historical, familial, physical, laboratory, behavioral, physiological, molecular imaging, and neuroimaging data and to perform a preventive intervention focusing on the disease, referred to as pre-emptive medicine. We refer to this predictive diagnostic method as ‘predictive differential diagnosis’ [24, 80]. To establish these preventive strategies, future well-designed, prospective cohort studies involving a large number of participants in several countries are essential. Because chronic fatigue is a contributing factor to various diseases [187–189], it should be a key target condition for pre-emptive medicine. This pre-emptive medicine may be a promising and strong strategy for health promotion.

In this review, we showed the frontier on fatigue, autonomic nerve dysfunction, and sleep-rhythm disorder, primarily based on the results of recent behavioral, neurophysiological, and neuroimaging studies. These findings provide new perspectives on the mechanisms underlying fatigue and on overcoming it, although future studies are needed.
